# Antennal transcriptomic analysis of carboxylesterases and glutathione S-transferases associated with odorant degradation in the tea gray geometrid, *Ectropis grisescens* (Lepidoptera, Geometridae)

**DOI:** 10.3389/fphys.2023.1183610

**Published:** 2023-04-04

**Authors:** Fangmei Zhang, Yijun Chen, Xiaocen Zhao, Shibao Guo, Feng Hong, Yanan Zhi, Li Zhang, Zhou Zhou, Yunhui Zhang, Xuguo Zhou, Xiangrui Li

**Affiliations:** ^1^ College of Agriculture, Xinyang Agriculture and Forestry University, Xinyang, China; ^2^ State Key Laboratory for Biology of Plant Diseases and Insect Pests, Institute of Plant Protection, Chinese Academy of Agricultural Sciences, Beijing, China; ^3^ College of Agriculture, Xinjiang Agricultural University, Urumqi, China; ^4^ Department of Entomology, University of Kentucky, Lexington, KY, United states

**Keywords:** *Ectropis grisescens*, antennal transcriptome, odorant-degrading enzyme, carboxylesterases, glutathione S-transferases

## Abstract

**Introduction:** Carboxylesterases (CXEs) and glutathione S-transferases (GSTs) can terminate olfactory signals during chemosensation by rapid degradation of odorants in the vicinity of receptors. The tea grey geometrid, *Ectropis grisescens* (Lepidoptera, Geometridae), one of the most devastating insect herbivores of tea plants in China, relies heavily on plant volatiles to locate the host plants as well as the oviposition sites. However, CXEs and GSTs involved in signal termination and odorant clearance in *E. grisescens* remains unknown.

**Methods:** In this study, identification and spatial expression profiles of CXEs and GSTs in this major tea pest were investigated by transcriptomics and qRT-PCR, respectively.

**Results:** As a result, we identified 28 CXEs and 16 GSTs from female and male antennal transcriptomes. Phylogenetic analyses clustered these candidates into several clades, among which antennal CXEs, mitochondrial and cytosolic CXEs, and delta group GSTs contained genes commonly associated with odorants degradation. Spatial expression profiles showed that most CXEs (26) were expressed in antennae. In comparison, putative GSTs exhibited a diverse expression pattern across different tissues, with one GST expressed specifically in the male antennae.

**Disscussion:** These combined results suggest that 12 CXEs (EgriCXE1, 2, 4, 6, 8, 18, 20-22, 24, 26, and 29) and 5 GSTs (EgriGST1 and EgriGST delta group) provide a major source of candidate genes for odorants degradation in *E. grisescens*.

## 1 Introduction

Insects have a highly specific and sensitive chemosensory system, which is extremely critical for sensing various chemical signals and regulating a series of behaviors (Elgar et al., 2018; Fleischer et al., 2018; Kang et al., 2021). Olfactory perception involves various proteins, including odorant binding proteins (OBPs), chemosensory proteins (CSPs), odorant receptors (ORs), gustatory receptors (GRs), ionotropic receptors (IRs), sensory neuron membrane proteins (SNMPs), and odorant degrading enzymes (ODEs) (Fleischer et al., 2018; Sun et al., 2020; Liu et al., 2021). Briefly, odorant molecules are bound and transported by OBPs onto ORs, and then ORs are activated, leading to signal transduction. Soon after, odor molecules are rapidly degraded by various ODEs to terminate the stimulation, ensuring that insect olfactory sensing systems keep stability and sensitivity for odor identity (Leal, 2013; Suh et al., 2014; Li et al., 2018; He et al., 2019).

ODEs, as multiple enzyme families expressed in the sensillar lymph, include carboxylesterases (CXEs) (Liu et al., 2019; Yi et al., 2021), glutathione S-transferases (GSTs) (Liu et al., 2021; Xia et al., 2022), cytochrome P450 monooxygenases (P450s) (Baldwin et al., 2021; Blomquist et al., 2021), UDP-glucuronosyltransferases (UGTs) (Zhang et al., 2017a), alcohol dehydrogenases (ADs) (Huang et al., 2016), and aldehyde oxidases (AOXs) (Zhang et al., 2017b; Wang et al., 2017). The first identified ODE, ApolPDE, a kind of CXE, could effectively degrade sex pheromone E6Z11-160Ac in *Antheraea polyphemus* (Vogt and Riddiford, 1981). Antennal-specific GSTs could quickly remove or degrade the odorants from ORs to maintain chemoreceptor sensitivity (Younus et al., 2014; Durand et al., 2018). The antennal-enriched P450s could degrade plant volatiles, insecticides, and pheromones (Cano-Ramirez et al., 2013; Keeling et al., 2013). Antennal ADs have roles in olfaction, which further necessitated investigating its odorant degradation function (Huang et al., 2016). *In vitro* functional studies clarified the odorant inactivation role of antennal AOXs, such as degrading sex pheromones and plant volatile aldehydes in *Amyelois transitella* (Choo et al., 2013).

Of these ODEs, CXEs and GSTs are the most well-studied and involved in degrading pheromone/odorant degradation and harmful volatile xenobiotics to maintain the sensitivity of the olfactory receptor neurons (ORNs). So far, many insect CXEs and GSTs have been identified, including *Chilo suppressalis* (Liu et al., 2015a), *Drosophila melanogaster* (Chertemps et al., 2015), *Ectropis obliqua* (Sun et al., 2017), *Spodoptera littoralis* (Durand et al., 2018), *Spodoptera exigua* (He et al., 2019), *Plodia interpunctella* (Liu et al., 2019; Liu et al., 2021), *Holotrichia parallela* (Yi et al., 2021), and *Sitophilus zeamais* (Xia et al., 2022), and their functions in insect olfactory perception have been characterized.

CXEs as multifunctional enzymes widely exist in insects, microbes, and plants (Guo and Wong, 2020). In insects, most CXEs are involved in detoxification of exogenous chemicals and are responsible for insecticide resistance and metabolic resistance (Mao et al., 2021). CXEs commonly share conserved active residues, such as the pentapeptide “G-X-S-X-G” (X represents any amino acid), oxyanion hole, glutamate (E), and histidine (H) residues (Godoy et al., 2021). Insect antennal CXEs are characterized as ODEs because they occur in the sensilla and can inactivate odor. They could be divided into ten major clades: mitochondrial and cytosolic esterases, dipteran microsomal α-esterases, cuticular and antennal esterases, β-esterases and pheromone esterases, Lepidopteran juvenile hormone esterases (JHEs), non-Lepidopteran JHEs, moth antennal esterases, neuroligins, neuroreceptors, and gliotactins (Durand et al., 2010a; Oakeshott et al., 2015). To date, many insect antennae CXEs have been identified and functionally characterized for their involvement in the degradation of pheromones or/and plant volatiles. For example, in the genus Spodoptera, two CXEs from *S. littoralis* (SlCXE7 and 10) (Durand et al., 2010b; Durand et al., 2011) and three from *S. exigua* (SexiCXE4, 10, and 14) (He et al., 2014a; He et al., 2014b; He et al., 2015) were functionally characterized and degraded both sex pheromones and plant volatiles. A similar phenomenon was also observed in *D. melanogaster* (EST6) (Chertemps et al., 2012; Chertemps et al., 2015) and *Plutella xylostella* (PxylCCE16c) (Wang et al., 2021). Additionally, a previous study indicated that CXEs modulated insect mating and foraging behaviors through inactivation of sex pheromones and host volatiles, which made them a novel target for pest behavioral inhibition. For example, the knockdown of GmolCXE1 and 5 had an impact on the mating behaviors of male moths of *Grapholita molesta* (Wei et al., 2020). Another CXE gene jhedup (duplication of the Juvenile hormone esterase gene) regulated the electrophysiological response and food-seeking ability by hydrolyzing various ester odorants of jhedup mutant *D. melanogaster* (Steiner et al., 2017).

Similar to CXEs, GSTs also are a diverse family of multifunctional enzymes with conserved amino-terminal domain and carboxyl-terminal domain, which have conserved GSH-binding site (G-site) and hydrophobic substrate (H-site), respectively (Ketterman et al., 2011; Durand et al., 2018). Insect GSTs are divided into six subclasses: delta, epsilon, omega, sigma, theta, and zeta, with some GSTs remaining unclassified, of which the delta- and epsilon-class GSTs are insect-specific (Shi et al., 2012; Labade et al., 2018). Insect GSTs are implicated in the detoxification of xenobiotic compounds (Huang et al., 2011). However, GST expression and their activities demonstrated that they cause signal termination within the olfactory organs (Durand et al., 2018; Tang et al., 2019). For example, in *G. molesta*, the biochemical characterization of GmolGSTD1 had confirmed its high degradation preference for pheromone component (Z)-8-dodecenyl alcohol and the host plant volatile butyl hexanoate, which showed that it could inactivate odorant molecules and maintain sensitivity to olfactory communication of *G. molesta* (Li et al., 2018). The antenna-highly expressed CpomGSTd2 in the codling moth, *Cydia pomonella*, interfered with odorant detection by depredating the odorant (Huang et al., 2017). The antenna-specific SzeaGSTd1 in *S. zeamais* also played a crucial role in host location by degrading the host volatile, capryl alcohol (Xia et al., 2022).

*Ectropis grisescens* (Lepidoptera, Geometridae), one of the most destructive pests in tea plantations, causing serious economic losses, is more widely distributed in major tea plantations in China than its counterparts (*E. obliqua* Prout) (Zhang et al., 2014; Zhang et al., 2016; Li et al., 2019). At present, control of this pest mainly depends on chemical insecticides, leading to environmental pollution and pest resistance (Pan et al., 2021). Moreover, pesticide residues affect the safety of drinking tea (Cao et al., 2018), so using insecticides is forbidden on organic tea plantations (Ma et al., 2016). Therefore, development of novel, effective, and environmentally friendly strategies to control this pest is urgently needed. ODEs thus play an important role in the termination of odorant signals and allow restoration of sensitivity of the olfactory system (Li et al., 2018; He et al., 2019). *E. grisescens* mainly depends on plant volatiles to search for host plants and locate oviposition sites. Thus, analyses of its mechanism of signal termination and odorant clearance in these important behaviors are necessary. Previous studies considered antennae CXEs or GSTs as potential molecular targets for developing novel pest management strategies based on the manipulation of chemoreception-driven behaviors (Wei et al., 2020; Xia et al., 2022). However, there is no relative report about CXEs or GSTs in *E. grisescens* yet.

In the present study, we sequenced and analyzed the antennal transcriptome of *E. grisescens* using Illumina sequencing. Then, CXE and GST gene families and subfamilies were identified and cloned; sequence architecture and phylogenetic analysis were carried out; and finally, quantitative real-time PCR (qRT-PCR) was used to profile their expression patterns in various tissues from both sexes. In addition, the potential roles of the identified CXEs and GSTs in signal termination of olfaction or other physiological processes were discussed. Our study on antennae-specific CXEs and GSTs is particularly important for understanding the molecular mechanism underlying odorant inactivation and subsequent development of ODE-based pest control strategies in *E. grisescens*.

## 2 Materials and methods

### 2.1 Insect rearing and sample collection

*Ectropis grisescens* larvae were collected from Mount Zhenlei (32°37′N, 114°42′E), Xinyang, Henan, China, and cultivated on fresh tea leaves in the laboratory under the constant conditions of 24°C ± 1°C, 65% relative humidity, and a 16:8 h L: D photoperiod. Emerged adults were fed with 10% honey solution. Antenna, head (without antennae), thorax, abdomen, wing, and leg tissues from 2-day-old unmated male and female insects were dissected, frozen in liquid nitrogen immediately, and stored at −80°C in a refrigerator.

### 2.2 RNA extraction, cDNA library construction, and sequencing

Total RNA was extracted from male and female antennae (n = 50, three replicates, respectively) using TRIzol reagent (Life Technologies, Carlsbad, CA, United States). The concentration of RNA samples was determined using a NanoDrop ND-1000 spectrophotometer (Thermo Scientific, Wilmington, DE, United States). RNA integrity was assessed using the RNA Nano 6000 Assay Kit of the Bioanalyzer 2100 system (Agilent Technologies, CA, United States). The cDNA libraries were constructed from RNA samples for Illumina sequencing following the Illumina protocol. Sequencing was carried out on the Illumina Novaseq platform (Novogene Co., Ltd., Beijing), and 150-bp paired-end reads were generated.

### 2.3 Sequence assembly and functional annotation

Raw data (raw reads) of FASTQ format were first modified into clean data (clean reads) through in-house Perl scripts, and clean data (clean reads) were obtained by removing reads containing adapter, reads containing ploy-N, and low-quality reads from raw data. At the same time, Q20, Q30, and GC content of the clean data was calculated. All the downstream analyses were based on the clean data with high quality.

Transcriptome assembly was performed using Trinity with min_kmer_cov set to 2 (Grabherr et al., 2011) by default and all other parameters set to default. Unigene functions were annotated based on NCBI NR, NT, KO, Swiss-Prot, Pfam, GO, and KEGG using Blastx and Blastn searches (E-value < 10^−5^), retrieving proteins with the highest sequence similarity for each transcript and their protein functional annotations. KEGG Automatic Annotation Server (KASS) was used to search KEGG with E-value = 10^−10^ (Götz et al., 2008), and Blast2GO v2.5 was used for GO annotation with E-value = 10^−6^ (Moriya et al., 2007).

Coding sequences (CDSs) were predicted through aligning transcriptome sequences to the NR and Swiss-Prot databases or using ESTScan 3.0.3 (Iseli et al., 1999). The read count for each gene was obtained by mapping clean reads to the assembled transcriptome using RSEM (Bowtie2 parameters: mismatch 0). The read count was calculated as Fragments Per Kilobase of transcript per Million mapped reads (FPKM) (Mortazavi et al., 2008).

### 2.4 Identification and bioinformatics analyses of candidate CXEs and GSTs

Candidate EgriCXEs and EgriGSTs were identified from the transcriptome data. Furthermore, all candidate EgriCXEs and EgriGSTs were manually checked by the BLASTx program at the NCBI. The complete coding regions were predicted by ORF finder (https://www.ncbi.nlm.nih.gov/orffinder/). Putative signal peptides were predicted with SignalP 5.0 (http://www.cbs.dtu.dk/services/SignalP). The isoelectric point (pI) and molecular weight (Mw) were computed by the ExPASy tool (https://web.expasy.org/compute_pi/). Multiple sequence alignment of the EgriCXE protein was performed using the Clustal program in the Jalview (v2.11.20) software with default parameters (Waterhouse et al., 2009). Phylogenetic trees were constructed in MEGA 11.0 software using the neighbor-joining method with 1000-fold bootstrap resampling (Kumar et al., 2018). The phylogenetic tree image was created by EvolView online software (https://www.evolgenius.info/).

### 2.5 Tissue expression analysis by qRT-PCR

Total RNA was extracted separately from antennae (n = 50), heads (without antennae; n = 10), thoraxes (n = 5), abdomens (n = 5), wings (n = 5), and legs (n = 10) from both sexes using TRIzol reagent (Life Technologies, Carlsbad, CA, United States). cDNA was synthesized by using TransScript One-Step gDNA Removal and cDNA Synthesis SuperMix (Tiangen, Beijing, China) following the manufacturer’s instructions. Primers specific for EgriCXEs and EgriGSTs were designed using Primer Premier 5.0 software (Premier Biosoft International, Palo Alto, CA, United States; Supplementary Table S1) and synthesized by Sangon Biotech (Shanghai, China).

The reaction volume of 20 µL was prepared using TB Green Premix Ex Taq (Tli RNase H Plus) (TaKaRa, Beijing) by following instructions from the manual. qRT-PCR was conducted using an Applied Biosystems 7500 Fast Real-Time PCR System (Applied Biosystems, Carlsbad, CA) under the following conditions: 95°C for 30 s, then 40 cycles of 95°C for 5 s and 60°C for 34 s, last 95°C for 15 s, 60°C for 1 min, 95°C for 15 s followed by the melting curve analysis. Three biological replicates and three technical replications were carried out. The housekeeping gene glyceraldehyde-3-phosphate dehydrogenase (GAPDH) was used as an internal control to normalize the data.

### 2.6 Statistical analysis

The relative quantification was calculated by the comparative 2^−ΔΔCT^ method (Livak and Schmittgen, 2001). The significance of each candidate EgriCXEs and EgriGSTs among various tissues was determined using a one-way analysis of variance (ANOVA). The significances of EgriCXEs and EgriGSTs from different tissues between female and male adult insects were assessed using a two-sample *t*-test in SAS statistical software 9.2 (SAS Institute Inc., Cary, NC, United States), with thresholds set at a *p* < 0.05.

## 3 Results

### 3.1 Transcriptome analysis and assembly

**FIGURE 1:**
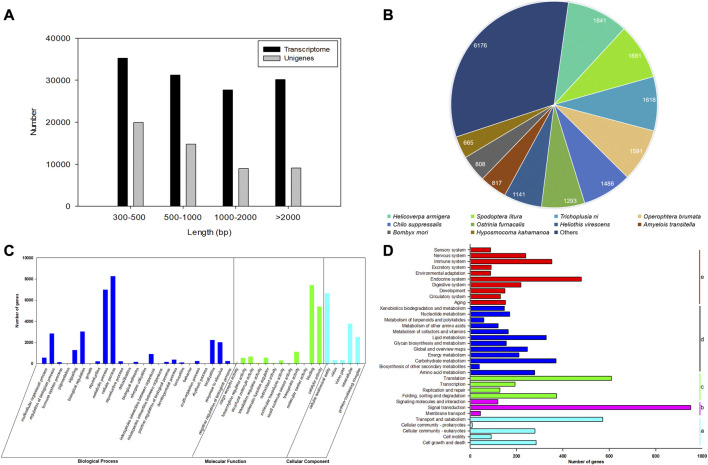
Characterization and transcriptome analysis of RNA sequences from antennae of *E. grisescens*. **(A)** Length distributions of the transcriptome and unigenes of *E. grisescens*. **(B)** Species distribution from the Blastx results of *E. grisescens* unigenes in the NR database. **(C)** GO classifications of *E. grisescens* unigenes. **(D)** KEGG classifications of *E. grisescens* unigenes; a: cellular processes, b: environmental information processing, c: genetic information processing, d: metabolism, e: organismal systems.

After filtering the low-quality and adapter reads, a total of 21,733,434 (97.27%), 23,300,358 (97.54%), 22,180,396 (96.87%), 22,713,765 (96.91%), 22,797,995 (98.15%), and 22,378,252 (97.27%) clean reads were generated from three replicates of female and male antennal libraries of *E. grisescens* (Supplementary Table S2). The total bases of sequence data were approximately 6.52–6.99 Gb from male and female samples. The average error rates of the sequences were 0.03%. The Q20 and Q30 values for each library exceeded 97% and 92%, respectively, with a GC content of 42.68%–45.79% (Supplementary Table S2). After merging and clustering, the 124,287 transcripts and 52,856 unigenes with a mean length of 1,518 and 1,233 bp, with N50 length of 2,552 and 2,170 bp, were identified, respectively (Supplementary Table S3). The length of transcriptomes and unigenes ranged from 301 to 30,857 bp, with an average length of 1,518 and 1,233 bp, respectively (Figure 1A, Supplementary Table S3).

### 3.2 Functional annotation

A total of 15,500 (29.32%), 7,321 (13.85%), 11,512 (21.77%), 14,325 (27.10%), 14,323 (27.09%), and 6,119 (11.57%) had BLASTn hits in the NT, KO, Swiss-Prot, PFAM, GO, and KOG databases, respectively (Supplementary Table S4). BLASTx results showed 19,118 (36.16%) unigenes had the best hits in the non-redundant protein (NR) database. Moreover, most of the annotated unigenes closely matched to Lepidoptera insect sequences (16,780), including *Helicoverpa armigera* (1,841), *Spodoptera litura* (1,681), *Trichoplusia ni* (1,618), *Heliothis virescens* (1,591), *C. suppressalis* (1,486), *Ostrinia furnacalis* (1,293), *H. virescens* (1,141), *Amyelois transitella* (817), *Bombyx mori* (808), and *Hyposmocoma kahamanoa* (665), as shown in Figure 1B.

Based on the GO annotations, 60,045 unigenes could be annotated into three functional categories: biological processes (50.37%), molecular function (26.98%), and cellular components (22.65%) (Figure 1C). A total of 42 GO terms were identified based on GO level 2, including the odorant recognition process, e.g., binding, catalytic activity, and transporter activity in the molecular function ontology, and localization, signaling, and response to stimulus in the biological process ontology. In the KEGG annotation, 7,936 unigenes were divided into five metabolic pathways: cellular processes, environmental information processing, genetic information processing, metabolism, and organismal systems. Most unigenes were assigned to signal transduction (11.98%), signaling molecules and interaction (1.51%), and environment adaptation (1.11%) involved in recognizing olfaction in insects (Figure 1D).

### 3.3 Identification of CXEs in *E. grisescens*

A total of 28 candidate EgriCXEs were identified from the antennal transcriptome of *E. grisescens*. The sequences were named EgriCXE1–6, 8–9, 11, 13, 15, 17–22, 24–27, 29–30, 32–34, and 36–37 according to their presumptive orthologs of *E. obliqua* (Sun et al., 2017) and were deposited in the GenBank database under accession numbers OQ296948 to OQ296975 (Table 1). All putative EgriCXEs shared relatively high identities (>60.93%) with their respective orthologs from other species, particularly its counterpart *E. obliqua*. The amino acid identity between these EgriCXEs ranged from 7.84% to 60.11% (Supplementary Table S5). EgriCXE sequences encoded 401 to 620 amino acid residues with molecular weight ranging from 45.25 to 69.63 kDa and the pI from 5.12 to 9.60. Furthermore, 11 EgriCXEs (EgriCXE1–2, 4–6, 13, 19, 21–22, 29, and 33) were predicted to have putative N-terminal signal peptides (Table 1). Multiple sequence alignment analyses showed that a conserved pentapeptide Gly–X–Ser–X–Gly motif (“X” represents any residue) and more variable oxyanion hole residues (Gly–Gly–Ala), conserved serine (S) residues, and glutamate (E) and histidine (H) residues of the catalytic triad were found (Supplementary Table S6).

**TABLE 1:** CXE identification and bioinformatics analysis of *E. grisescens* antennal transcriptomes.

Gene name	GenBank accession	ORF (aa)	SP	MW (KDa)	pI	BLASTX best hit		
						(Name/species)	Accession number	Identity (%)
EgriCXE1	OQ296948	565	Yes	63.56	7.17	Putative antennal esterase CXE1 [*Ectropis obliqua*]	ARM65372.1	98.05
EgriCXE2	OQ296949	518	Yes	59.51	6.14	Putative antennal esterase CXE2 [*Ectropis obliqua*]	ARM65373.1	97.30
EgriCXE3	OQ296950	535	No	59.94	5.94	Putative antennal esterase CXE3 [*Ectropis obliqua*]	ARM65374.1	99.07
EgriCXE4	OQ296951	516	Yes	57.92	8.30	Putative antennal esterase CXE4 [*Ectropis obliqua*]	ARM65375.1	97.68
EgriCXE5	OQ296952	595	Yes	66.35	6.65	Putative antennal esterase CXE5 [*Ectropis obliqua*]	ARM65376.1	98.15
EgriCXE6	OQ296953	558	Yes	60.95	5.36	Putative antennal esterase CXE6 [*Ectropis obliqua*]	ARM65377.1	98.92
EgriCXE8	OQ296954	429	No	48.31	6.03	Putative antennal esterase CXE8 [*Ectropis obliqua*]	ARM65379.1	57.24
EgriCXE9	OQ296955	558	No	64.01	7.54	Putative antennal esterase CXE9 [*Ectropis obliqua*]	ARM65380.1	97.67
EgriCXE11	OQ296956	523	No	58.24	5.89	Putative antennal esterase CXE11 [*Ectropis obliqua*]	ARM65382.1	96.75
EgriCXE13	OQ296957	560	Yes	62.12	6.32	Putative antennal esterase CXE13 [*Ectropis obliqua*]	ARM65384.1	98.57
EgriCXE15	OQ296958	401	No	45.25	9.60	Putative antennal esterase CXE15 [*Ectropis obliqua*]	ARM65386.1	98.58
EgriCXE17	OQ296959	495	No	56.05	5.81	Putative antennal esterase CXE17 [*Ectropis obliqua*]	ARM65388.1	97.72
EgriCXE18	OQ296960	542	No	61.16	6.43	Putative antennal esterase CXE18 [*Ectropis obliqua*]	ARM65389.1	99.08
EgriCXE19	OQ296961	609	Yes	69.13	5.30	Putative antennal esterase CXE19 [*Ectropis obliqua*]	ARM65390.1	99.84
EgriCXE20	OQ296962	501	No	56.55	6.13	Putative antennal esterase CXE20 [*Ectropis obliqua*]	ARM65391.1	97.92
EgriCXE21	OQ296963	564	Yes	62.68	5.37	Putative antennal esterase CXE21 [*Ectropis obliqua*]	ARM65392.1	98.70
EgriCXE22	OQ296964	568	Yes	63.42	5.93	Putative antennal esterase CXE22 [*Ectropis obliqua*]	ARM65393.1	97.71
EgriCXE24	OQ296965	567	No	63.76	8.70	Putative antennal esterase CXE24 [*Ectropis obliqua*]	ARM65395.1	97.88
EgriCXE25	OQ296966	570	No	64.10	8.72	Putative antennal esterase CXE25, partial [*Ectropis obliqua*]	ARM65396.1	97.71
EgriCXE26	OQ296967	523	No	59.26	6.02	Putative antennal esterase CXE26 [*Ectropis obliqua*]	ARM65397.1	99.24
EgriCXE27	OQ296968	567	No	53.99	6.53	Putative antennal esterase CXE27 [Ectropis obliqua]	ARM65398.1	95.83
EgriCXE29	OQ296969	569	Yes	63.78	5.83	Putative antennal esterase CXE29 [*Ectropis obliqua*]	ARM65400.1	96.74
EgriCXE30	OQ296970	438	No	49.61	5.50	Putative antennal esterase CXE30 [*Ectropis obliqua*]	ARM65401.1	98.59
EgriCXE32	OQ296971	620	No	65.60	6.42	Putative antennal esterase CXE32 [*Ectropis obliqua*]	ARM65403.1	99.22
EgriCXE33	OQ296972	555	Yes	62.35	5.12	Putative antennal esterase CXE33 [*Ectropis obliqua*]	ARM65404.1	99.02
EgriCXE34	OQ296973	563	No	63.95	5.38	Putative antennal esterase CXE34 [*Ectropis obliqua*]	ARM65405.1	98.76
EgriCXE36	OQ296974	532	No	59.13	5.42	Esterase FE4 [*Bombyx mori*]	XP_004932947.1	64.98
EgriCXE37	OQ296975	615	No	69.63	6.69	Carboxylesterase 1C [*Helicoverpa armigera*]	XP_021188868.2	60.93

### 3.4 Identification of GSTs in *E. grisescens*

A total of 16 candidate EgriGSTs were identified and named EgriGSTe1 to EgriGSTu1, and they were deposited in the GenBank database under the accession numbers OQ296976 to OQ296991 (Table 2). EgriGSTs encoded 179 to 298 amino acid residues, with molecular weight ranging from 20.06 to 33.67 kDa and the pI from 4.95 to 9.46. Blastx search of the best hits showed that all EgriGST sequences shared relatively high sequence identities (53.14%–100.00%) with their respective orthologs from other insects (Table 2). The sequence identities of these EgriGSTs range from 5.50% to 86.76% (Supplementary Table S7). Multiple sequence alignment analyses of the EgriGSTs showed that a conserved G-site can be found in the N-terminal domain and a more variable H-site can be observed with a low sequence identity in the C-terminal domain (Supplementary Figure S1).

**TABLE 2:** GST identification and bioinformatics analysis of *E. grisescens* antennal transcriptomes.

Clade	Gene name	GenBank accession	ORF (aa)	MW (KDa)	pI	BLASX best hit		
						Name/species	Accession number	Identity (%)
Epsilon	EgriGSTe1	OQ296976	179	19.69	4.64	Glutathione S-transferase 1 isoform X2 [*Helicoverpa armigera*]	XP_021180950.2	55.93
	EgriGSTe2	OQ296977	229	26.22	8.74	Glutathione S-transferase 1-like [*Galleria mellonella*]	XP_031763098.1	69.33
	EgriGSTe3	OQ296978	179	20.06	5.57	Glutathione S-transferase 10 [*Streltzoviella insularis*]	QLI62206.1	55.31
	EgriGSTe4	OQ296979	218	25.11	5.83	Glutathione S-transferase 1-like [*Bombyx mandarina*]	XP_028040596.1	53.14
	EgriGSTe5	OQ296980	241	27.64	6.52	Glutathione S-transferase epsilon class [*Spodoptera littoralis*]	AYM01167.1	66.18
Delta	EgriGSTd1	OQ296981	215	24.16	4.95	Glutathione S-transferase 1-1-like [*Pectinophora gossypiella*]	XP_049871107.1	65.28
	EgriGSTd2	OQ296982	216	24.23	6.17	Glutathione S-transferase 1-like [*Bombyx mandarina*]	XP_028025239.1	79.17
	EgriGSTd3	OQ296983	298	33.67	9.46	Glutathione S-transferase delta 3 [*Ostrinia furnacalis*]	QIC35739.1	64.13
	EgriGSTd4	OQ296984	216	24.25	6.44	Glutathione S-transferase 1 [*Manduca sexta*]	XP_030035460.2	83.80
Sigma	EgriGSTs1	OQ296985	204	24.06	5.50	Glutathione S-transferas-like [*Pectinophora gossypiella*]	XP_049871113.1	60.20
	EgriGSTs2	OQ296986	219	25.14	6.34	Glutathione S-transferase 2-like [*Manduca sexta*]	XP_030022570.1	67.96
	EgriGSTs3	OQ296987	218	25.97	7.72	Glutathione S-transferase Mu 1 isoform 2 [*Mus musculus*]	NP_034488.1	100.00
	EgriGSTs4	OQ296988	204	23.96	5.71	Glutathione S-transferase sigma 3 [*Operophtera brumata*]	KOB62848.1	63.18
Theta	EgriGSTt1	OQ296989	228	26.41	7.76	Glutathione S-transferase theta 1 [*Bombyx mori*]	NP_001108463.1	71.62
Omega	EgriGSTo1	OQ296990	104	12.09	5.07	Glutathione S-transferase omega 1 [*Heortia vitessoides*]	AWX68890.1	90.48
Unclassified	EgriGSTu1	OQ296991	231	26.30	5.73	Unclassified glutathione S-transferase [*Chilo suppressalis*]	AKS40352.1	78.26

### 3.5 Phylogenetic analysis

**FIGURE 2:**
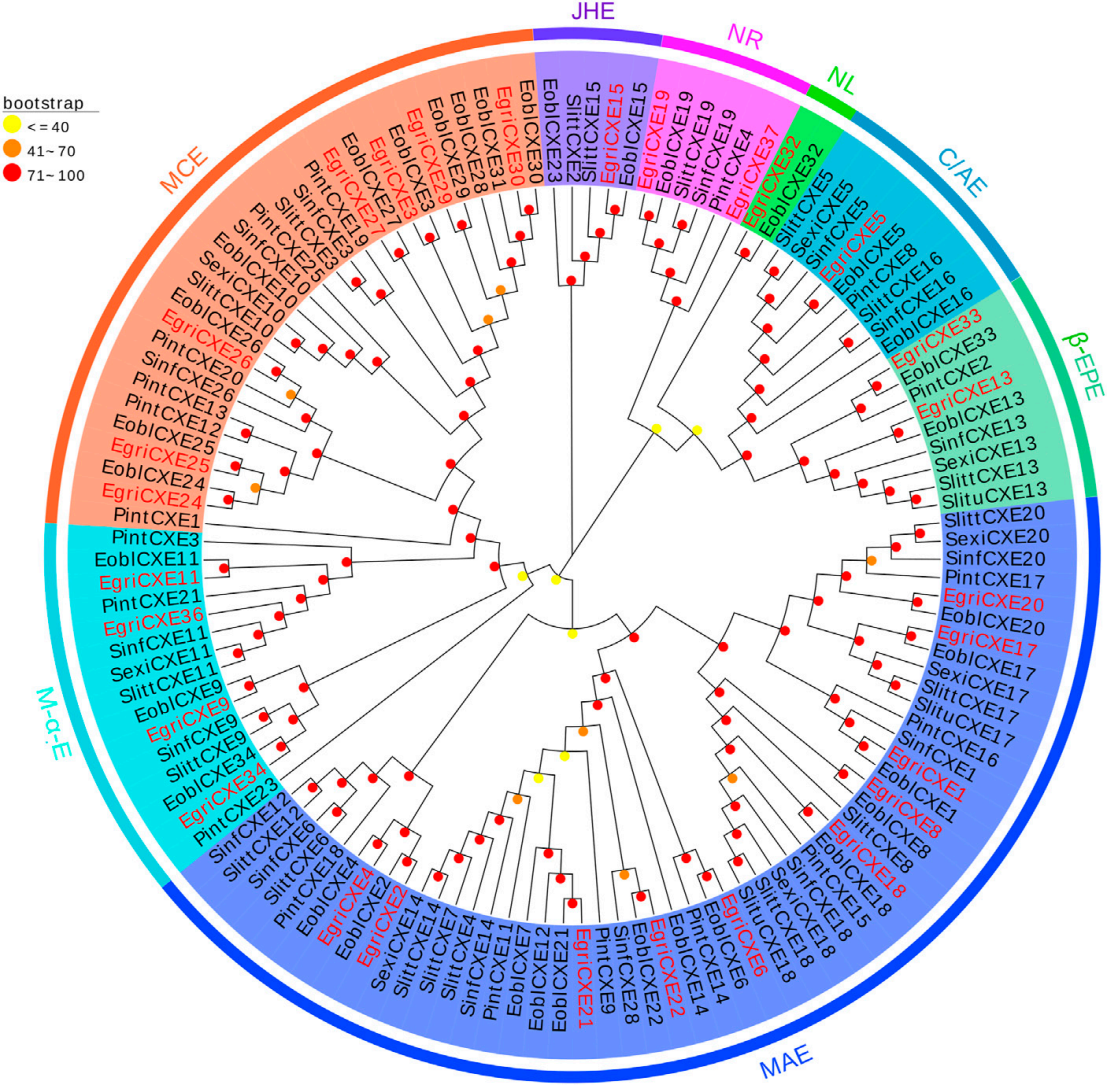
Phylogenetic analysis of candidate EgriCXEs with other insect CXEs. MCEs: mitochondrial and cytosolic esterases; JHEs: Lepidopteran juvenile hormone esterases; NR: neuroreceptor; NL: neuroligin; C/AEs: cuticular and antennal esterases; β-EPEs: β-esterases and pheromone esterases; MAEs: moth antennal esterases; M-α-Es: microsomal α-esterases. Egri, *Ectropis grisescens* (N = 28); Eobl, *Ectropis obliqua* (N = 34); Slitt, *Spodoptera littoralis* (N = 19); Pint, *Plodia interpunctella* (N = 19); Sexi, *Spodoptera exigua* (N = 8); Slitu, *Spodoptera litura* (N = 3); Sinf, *Sesamia inferens* (N = 16). Candidate EgriCXEs are indicated by red. The GenBank accession numbers of the 127 CXEs protein used in the phylogenetic analysis are listed in Supplementary Table S8.

The phylogenetic tree of EgriCXEs was constructed with 127 CXE sequences from six kinds of Lepidoptera insects: *E. obliqua*, *S. littoralis*, *P. interpunctella*, *S. exigua*, *S. litura*, and *Sesamia inferens* (Figure 2). The results showed that EgriCXEs were distributed in eight different clades: 1) EgriCXE1, 2, 4, 6, 8, 17–18, and 20–22 were clustered with the moth antennal esterase group; 2) EgriCXE13 and 33 were assigned into β-esterase and pheromone esterase group; 3) EgriCXE5 and 4) EgriCXE32 were distributed into cuticular and antennal esterases and neuroligins, respectively; 5) two EgriCXEs (EgriCXE9 and 37) and 6) four EgriCXEs (EgriCXE 9, 11, 34, and 36) were assigned into neuroreceptor and microsomal α-esterases, respectively; 7) EgriCXE3, 24–27, 29, and 30 were clustered with mitochondrial and cytosolic esterases; 8) EgriCXE15 was assigned into Lepidopteran juvenile hormone esterases.

**FIGURE 3:**
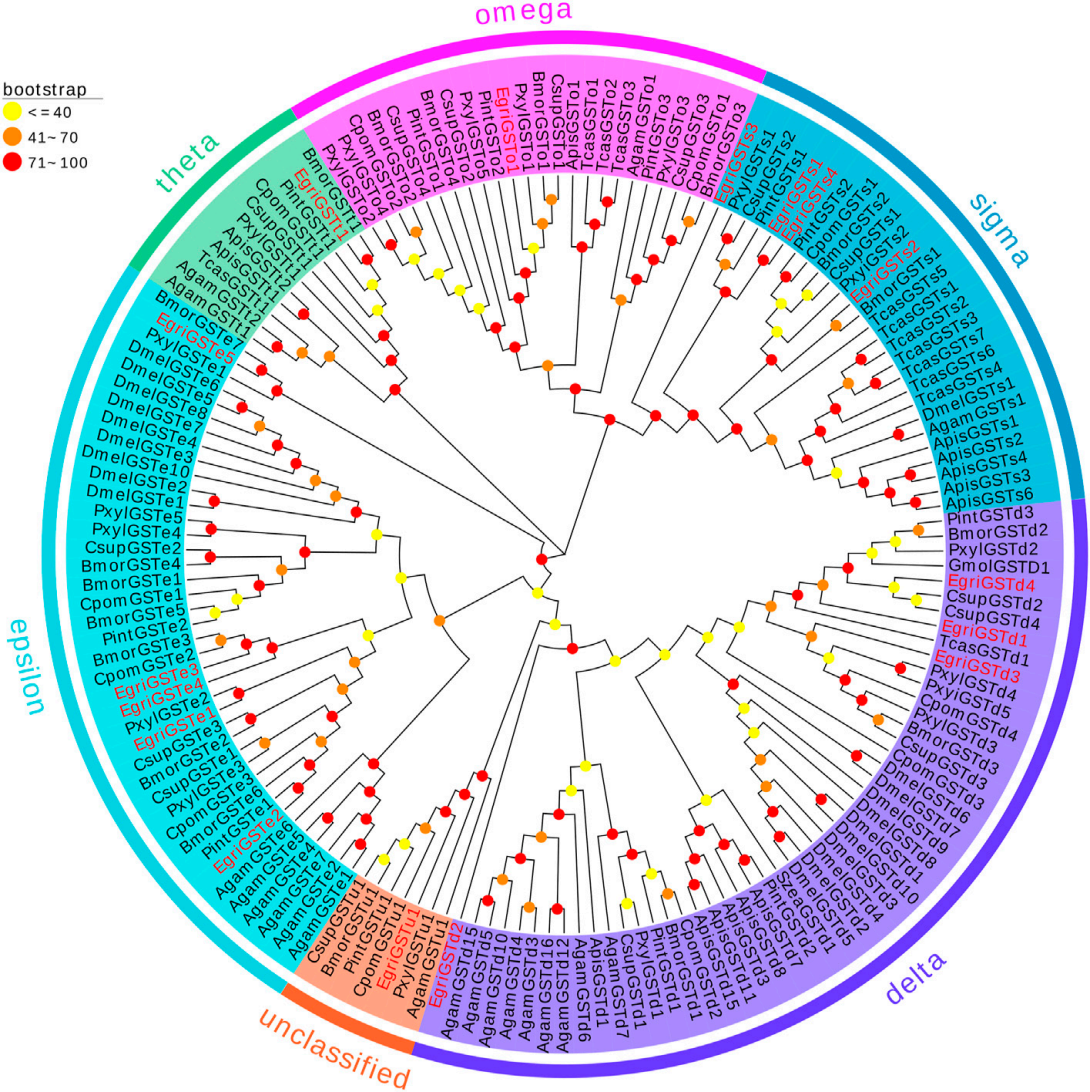
Phylogenetic analysis of candidate EgriGSTs with other insect GSTs. Egri, *Ectropis grisescens* (N = 16); Pint, *Plodia interpunctella* (N = 12); Pxyl, *Plutella xylostella* (N = 19); Cpom, *Cydia pomonella* (N = 11); Bmor, *Bombyx mori* (N = 18); Csup, *Chilo suppressalis* (N = 15); Apis, *Acyrthosiphon pisum* (N = 14); Dmel, *Drosophila melanogaster* (N = 20); Agam, *Anopheles gambiae* (N = 16); Tcas, *Tribolium castaneum* (N = 12); Gmol, *Grapholita molesta* (N = 1); Szea, *Sitophilus zeamais* (N = 1). Candidate EgriGSTs are indicated by red. The GenBank accession numbers of the 159 GSTs protein used in the phylogenetic analysis are listed in Supplementary Table S9.

The phylogenetic tree of EgriGSTs was constructed with 159 GST sequences from eleven insect species: *P. interpunctella*, *P. xylostella*, *C. pomonella*, *B. mori*, *C. suppressalis*, *Acyrthosiphon pisum*, *D. melanogaster*, *Anopheles gambiae*, *Tribolium castaneum*, *G. molesta*, and *S. zeamais* (Figure 3). The results showed that the EgriGSTs were divided into six different GST groups: 1) five epsilon EgriGSTs (EgriGSTe1–5); 2) four delta EgriGSTs (EgriGSTd1–4); 3) four sigma EgriGSTs (EgriGSTs1–4); 4) a theta EgriGSTt1; 5) an omega EgriGSTo1, and 6) an unclassified EgriGSTu1.

### 3.6 Expression profiles of EgriCXEs and EgriGSTs

**FIGURE 4:**
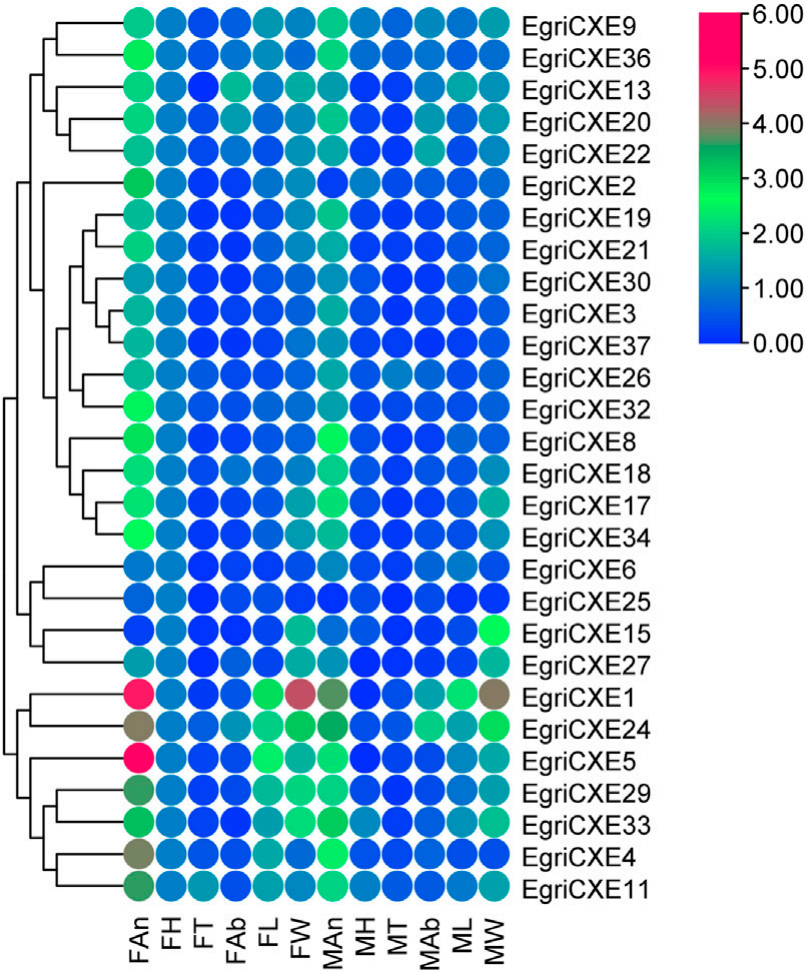
Tissue- and sex-specific expression pattern of the EgriCXEs. A total of 28 EgriCXEs were clustered using relative expression values from each of the tissues. Expression values are relative to female head tissue (one-fold), with red and blue representing the highest and the lowest values, respectively. FAn: female antennae; FH: female head; FT: female thorax; FAb: female abdomen; FL: female leg; FW: female wing; MAn: male antennae; MH: male head; MT: male thorax; MAb: male abdomen; ML: male leg; MW: male wing.

qRT-PCR results of EgriCXEs (Figure 4) in antennae, heads, thoraxes, abdomens, legs, and wings of both sexes showed that 26 EgriCXEs (*EgriCXE1–6*, *8–9*, *11*, *13*, *17–22*, *24*, *26–27*, *29–30*, *32–34*, and *36–37*) displayed significant antennal bias expression pattern, except for *EgriCXE15* and *EgriCXE25*. Of them, *EgriCXE1–2*, *4*, *8*, *11*, *13*, *18*, *20–22*, *24*, *26*, *29*, *32*, *34*, and *36–37* were significantly highly expressed in female antennae compared to the male antennae (*t*_*EgriCXE1*_ = 17.21, *p* < 0.0001; *t*_*EgriCXE2*_ = 38.90, *p* < 0.0001; *t*_*EgriCXE4*_ = 12.52, *p* = 0.0002; *t*_*EgriCXE8*_ = 7.20, *p* = 0.0020; *t*_*EgriCXE11*_ = 9.48, *p* = 0.0007; *t*_*EgriCXE13*_ = 12.77, *p* = 0.0002; *t*_*EgriCXE18*_ = 4.67, *p* = 0.0095; *t*_*EgriCXE20*_ = 3.71, *p* = 0.0207; *t*_*EgriCXE21*_ = 5.06, *p* = 0.0072; *t*_*EgriCXE22*_ = 3.28, *p* = 0.0304; *t*_*EgriCXE24*_ = 5.66, *p* = 0.0048; *t*_*EgriCXE26*_ = 4.67, *p* = 0.0095; *t*_*EgriCXE29*_ = 19.63, *p* < 0.0001; *t*_*EgriCXE32*_ = 10.92, *p* = 0.0004; *t*_*EgriCXE3*4_ = 8.03, *p* = 0.0013; *t*_*EgriCXE3*6_ = 7.10, *p* = 0.0021; and *t*_*EgriCXE37*_ = 8.49, *p* = 0.0011). Only *EgriCXE6* were highly expressed in male antennae, showing male-specific expression (*t*_*EgriCXE6*_ = −8.02, *p* = 0.0013). Other EgriCXEs (*EgriCXE3*, *5*, *9*, *17*, *19*, *27*, *30,* and *33*) showed no significant difference between the two sexes (*t*_*EgriCXE3*_ = −2.08, *p* = 0.1061; *t*_*EgriCXE5*_ = 1.98, *p* = 0.1857; *t*_*EgriCXE9*_ = 0.25, *p* = 0.8130; *t*_*EgriCXE17*_ = 1.49, *p* = 0.2115; *t*_*EgriCXE19*_ = −2.19, *p* = 0.0934; *t*_*EgriCXE27*_ = −0.45, *p* = 0.6873; *t*_*EgriCXE3*0_ = 2.16, *p* = 0.0966; and *t*_*EgriCXE33*_ = 2.14, *p* = 0.0989), whereas, *EgriCXE15* and *EgriCXE25* were highly expressed in a non-chemosensory organ, heads and wings of both sexes, respectively. Furthermore, all EgriCXE genes were detected in other non-chemosensory organs with less expression levels.

**FIGURE 5:**
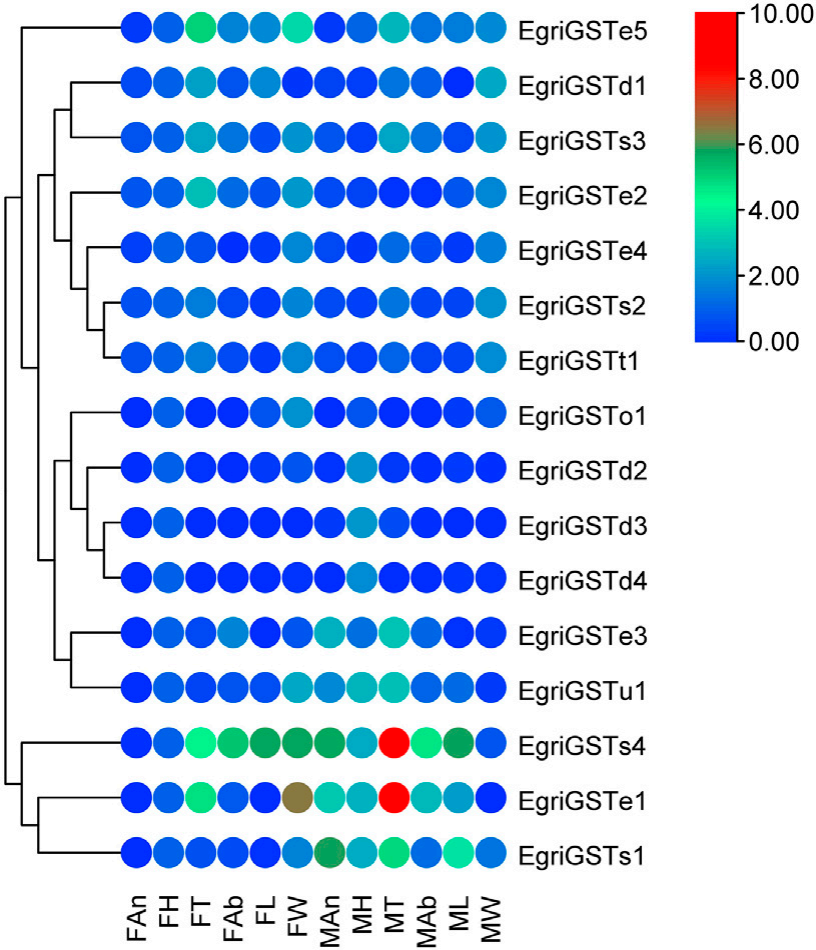
Tissue- and sex-specific expression pattern of the EgriGSTs. A total of 16 EgriGSTs were clustered using relative expression values from each of the tissues.

The putative EgriGSTs showed a wide range of expression patterns (Figure 5): *EgriGSTe1*, *s4*, and *u1* were highly expressed in male thoraxes and female wings, whereas *EgriGSTe2* and *d1* were strongly expressed in female thoraxes and male wings. *EgriGSTe3* was highly expressed in male thoraxes and female abdomens. *EgriGSTe4*, *s2*, *t1*, and *o1* were highly expressed in the wings of both sexes. *EgriGSTd2*, *d3*, and *d4* were strongly expressed in the heads of both sexes. *EgriGSTe5* and *s3* were strongly expressed in the thoraxes of both sexes. *EgriGSTs1* was specifically expressed in male antennae, with a significant difference between the two sexes (*t*_*EgriGSTs1*_ = −26.43, *p* = 0.0014). Some EgriGSTs were widely distributed and detected in various tissues, e.g., *EgriGSTs2* and *EgriGSTs3*.

## 4 Discussion

In the present research, we identified 52,856 unigenes with a mean length of 1,233 bp from the male and female *E. grisescens* antennal transcriptome, indicating the high quality and great depth of sequencing at the transcriptome level. The results of Blastx homology search in the NCBI database revealed that *E. grisescens* unigenes shared relatively high homology with sequences from other Lepidoptera species. Ultimately, 28 candidate CXE genes and 16 candidate GST genes were identified in the *E. grisescens* antennal transcriptome.

In GO annotation of the transcriptome, several annotations were associated with olfaction in insects such as binding, catalytic activity, and transporter activity in the molecular function ontology, localization, signaling, and response to stimulus in the biological process ontology, which are vital steps of odorant recognition in insects (Schmidt and Benton, 2020; Zhang et al., 2020; Rihani et al., 2021; Tian et al., 2021). KEGG pathway analysis also had similar function annotations about recognizing olfaction, such as signal transduction and environment adaptation. The aforementioned results of function annotation were similar to the finding in *Bemisia tabaci* MED (Wang et al., 2017) and *Athetis dissimilis* (Song et al., 2021), which further showed that the identified EgriCXEs and EgriGSTs might participate in various chemical communications of *E. grisescens*.

The number of CXE genes identified in *E. grisescens* was the same as that in *Cnaphalocrocis medinalis* (Zhang et al., 2017b) and *Athetis lepigone* (Zhang et al., 2017c). However, this number was significantly greater than that of CXE genes identified in other species reported, such as *C. pomonella* (12) (Huang et al., 2016), *C. suppressalis* (19) (Liu et al., 2015a), *H. parallela* (20) (Yi et al., 2021), and *A. lepigone* (20) (Zhang et al., 2017c). The difference in gene numbers among different species might depend on the evolution of divergent behaviors in the long term, which resulted in gene duplication and loss (Zhang et al., 2017b; Huang et al., 2021). In addition, the sample preparation and sequencing method/depth might also be a reason. Multiple sequence alignment analyses showed that most EgriCXEs had the oxyanion hole residues Gly–Gly–Ala, the catalytic triad Ser–Glu–His, and the conserved pentapeptide Gly–X–Ser-–X–Gly. These characteristics indicated that most of the identified EgriCXEs might encode functional enzymes and play a vital role in the catalytic activity of CXEs (Oakeshott et al., 2010).

The number of GST genes identified in *E. grisescens* was different from that of *S. zeamais* (13) (Xia et al., 2022), *P. interpunctella* (17) (Liu et al., 2021), *S. littoralis* (33) (Durand et al., 2018), and *C. medinalis* (23) (Liu et al., 2015b). This massive increase in the number of of GSTs in insects enables them to detect plant compounds and resist the damage caused by insecticides, as described in some reports (Durand et al., 2018; Liu et al., 2021). The results of the EgriGST sequence analysis showed a conserved G-site can be found in the N-terminal domain, indicating function as GSH-binding. However, a more variable H-site could be observed with a low sequence identity in the C-terminal domain, which enabled GSTs to accommodate various substrate selectivities (Lerksuthirat and Ketterman, 2008). Furthermore, we also found that some full-length EgriCXEs or EgriGSTs had low identities of amino acid sequences, which suggested that these genes evolved rapidly during long-term adaptation to various environmental factors (Campanini and De Brito, 2016).

The phylogenetic tree showed that the EgriCXEs could be divided into eight groups using classifications from the previous studies (Durand et al., 2010a; Oakeshott et al., 2015). The majority of the antennal EgriCXEs were assigned to the clade that contained members of the esterase family. Among them, ten EgriCXEs (EgriCXE1, 2, 4, 6, 8, 17–18, and 20–22) constituted the biggest groups, moth antennal esterase branch, together with the SexiCXE14 of *S. exigua* (He et al., 2014c), and SlittCXE7 of *S. littoralis* (Durand et al., 2011), which caused the degradation of plant volatiles and pheromone compounds. Of them, nine EgriCXEs (EgriCXE1, 2, 4, 6, 8, 18, and 20–22) were significantly expressed in female or male antenna, suggesting these CXEs might have a similar function in odorant degradation. Seven EgriCXEs (EgriCXE3, 24–27, 29, and 30) were clustered into mitochondrial and cytosolic esterase clades with two well-characterized CXEs, SlittCXE10 and SexiCXE10, which were specifically active to plant volatiles pentyl acetate and Z3-6: Ac, respectively (Durand et al., 2010b; He et al., 2015). Of them, three EgriCXEs (EgriCXE 24, 26, and 29) showed high antennal bias, indicating that these EgriCXEs were potentially involved in odorant degradation.

Additionally, two EgriCXEs (EgriCXE13 and 33), along with annotated pheromone and plant volatiles degrading enzymes (like SexiCXE13 for plant volatiles pentyl acetate and the acetate sex pheromone Z9E12-14: Ac, (He et al., 2014a)), were assigned to the β-esterase and pheromone esterase clades. However, the expression pattern of EgriCXE13 and 33 showed no antennal bias in our qRT-PCR. This phenomenon was also observed in the microsomal α-esterase clade, containing four EgriCXEs (EgriCXE9, 11, 34, and 36) without antennal bias and SexiCXE11 which had a high degradation activity against sex pheromones Z9-14: Ac and plant volatile esters pentyl acetate (He et al., 2019). Therefore, functional confirmation of these CXEs would require further functional analyses using *in vitro* and *in vivo* methods.

EgriCXE32 constituted Lepidopteran JHEs, which could control juvenile hormone (JH) titer and regulate larval to adult transition in insects (Gu et al., 2015). Two EgriCXEs (EgriCXE19 and 37) and EgriCXE5 were assigned into neuroreceptor and neuroligins clades, respectively, which were mainly involved in neurological and sensory developmental function (Durand et al., 2017). The cuticular and antennal esterase clade only contained one EgriCXE (EgriCXE15) without antennal-biased expression, and this gene lacked CXE conserved characteristic, the oxyanion hole-forming residues (Supplementary Table S6), indicating that it might play other roles, such as detoxification of insecticides.

The phylogenetic tree showed that the EgriGSTs could be divided into six groups. The epsilon class group was common GSTs in insects, which was widely recognized to have a detoxification function (Lalouette et al., 2016; Labade et al., 2018), mediating endocrine plasticity (Bigot et al., 2012) and cholesterol transporter activity (Enya et al., 2015). For instance, SlitGSTe1 and SlitGSTe2 in *S. littoralis* antennae were induced by sublethal doses of deltamethrin and were involved in protecting against insecticides (Lalouette et al., 2016). The expression pattern suggested EgriGSTe1–5 was highly expressed in thoraxes, abdomens, and wings, which further verified their function as degraders of non-volatile substances.

Additionally, GSTs of the insect delta group in antennae were commonly associated with odorant degradation, which had been studied and verified in some moths. For example, PintGSTd1 of *P. interpunctella* could efficiently degrade the sex pheromone component Z9-12: Ac and host volatile hexanal (Liu et al., 2021). GmolGSTD1 of *G. molesta* exhibited high degradation activity to the sex pheromone component (Z)-8-dodecenyl alcohol and the host plant volatile butyl hexanoate (Li et al., 2018). SzeaGSTd1 of *S. zeamais* could degrade the volatile of the host capryl alcohol (Xia et al., 2022). Unexpectedly, we found that none of the delta genes in *E. grisescens* was restricted to antenna-based qRT-PCR results, which were mainly expressed in the male heads or ubiquitously expressed in tissues tested. However, phylogenetic tree analysis showed that the delta genes in *E. grisescens* were distributed among the well-defined insect GST clades, together with GmolGSTD1, PintGSTd1, and SzeaGSTd1. Considering this point, we hypothesized that the delta group mediated the degradation of odorants in *E. grisescens*, which needed to be further investigated.

The sigma class genes had diverse functions, e.g., recognizing invasive pathogens (Huang et al., 2011) and detoxifying insecticides (Qin et al., 2012; Qin et al., 2013). The expression pattern showed that the sigma group genes in *E. grisescens* exhibited ubiquitous expression patterns, with the exception of EgriGSTs1, whose expression was almost restricted in the male antennae, suggesting that EgriGSTs1 might play a crucial role in inactivating the chemical signals or pheromone-degrading enzymes (PDEs). Other classes (theta, omega, and unclassified group) were involved in the detoxification of xenobiotics (Qin et al., 2013; Yamamoto and Yamada, 2016; Durand et al., 2018).

In conclusion, we characterized 28 carboxylesterases and 16 glutathione S-transferases encoding odorant-degrading enzymes (ODEs) from *E. grisescens* antennal transcriptome. Furthermore, the expression patterns of carboxylesterases and glutathione S-transferases in different tissues were investigated to identify antennal enriched genes. Finally, 12 EgriCXEs (EgriCXE1, 2, 4, 6, 8, 18, 20–22, 24, 26, and 29) and five GSTs (EgriGST1 and EgriGST delta group) were identified as candidate target genes involved in odorant degradation of *E. grisescens*. The findings of this study revealed the potential involvement of ODEs in the olfactory system of *E. grisescens*.

## Data Availability

The data presented in the study are deposited in the Sequence Read Archive repository, accession number PRJNA784387. The data link: https://www.ncbi.nlm.nih.gov/sra/?term=PRJNA784387.
