# Supplementary material for: Antennal transcriptomic analysis of carboxylesterases and glutathione S-transferases associated with odorant degradation in the tea gray geometrid, Ectropis grisescens (Lepidoptera, Geometridae)
Source: Front Physiol. 2023 Apr 4;14:1183610. doi: 10.3389/fphys.2023.1183610 (PMC10110894; doi:10.3389/fphys.2023.1183610)
Supplement: Supplementary file 3 [file Table1.DOCX]

Supplementary Material

Antennal transcriptomic analysis of carboxylesterases and glutathione S-transferases associated with odorant degradation in the tea grey geometrid, *Ectropis grisescens* (Lepidoptera, Geometridae)

Fangmei Zhang^1^, Yijun Chen^1, 2^, Xiaocen Zhao^1^, Shibao Guo^1^, Yanan Zhi^1^, Li Zhang^1^, Zhou Zhou^1^, Yunhui Zhang^3^, Xuguo Zhou^4^, Xiangrui Li^3, *^

*** Correspondence:** Xiangrui Li, xrli@ippcaas.cn

# Supplementary Tables

**Table S1. Primers used in this study**

| **Gene name** | **Forward primes (5’-3’)** | **Reverse primes (5’-3’)** |
| --- | --- | --- |
| EgriCXE1 | GCTTGACGCTGTGGACGAGAAC | GATGTAGACCATGACCGGGAGGAG |
| EgriCXE2 | GACGAGTGGTGAAGTTGTGGATGG | CGAAGCGAGTTCAGGTCTGTGG |
| EgriCXE3 | ACTCGTCGCAACTGACCCTACC | GTCTGAAGGTGCCTCCGTTATGAAG |
| EgriCXE4 | CGCAGAGCTATCCCAACTTCACTAC | AAGATGCCGCCTTGAGCTATAATCG |
| EgriCXE5 | CGACAACAGCTCCGCCATCATC | AACCCGAACTCATAGAACCCTCTCC |
| EgriCXE6 | TTAACCCTCAACAAGCCACGATGAC | CAACAGACAGTAGTCCGGCCATTC |
| EgriCXE8 | ACGACGACCTGATCTACCTGTTCTC | TGAAGTCTACCATGCGGGAGTCC |
| EgriCXE9 | TGCGAAATACGGGAACCCAACAC | TTCCGTCCCTTCGTTAATTCTGTGG |
| EgriCXE11 | CACGCTGACGATACGGGCTATG | TCACTGTCACTGGGAGTAGGATTGG |
| EgriCXE13 | TCCGCAGCAACATCCTCAACTTC | GTGGCGAGAGGTAGTGGTAATGAAC |
| EgriCXE15 | GCTACCACCTTGTATCACCCATGTC | TCTGAATGGCATCCGAGTTGTCTTG |
| EgriCXE17 | CTCCTGGAAACGCCGCTATGAAG | TCTCGCCGAAGAGTGTCACATTATC |
| EgriCXE18 | AGGGATTTTGAGCGGTAAAGTGAGC | GCCTTGAACCTCGTACTGCTGTTAG |
| EgriCXE19 | TCCACCAACGTCGAATGAAACCG | CGTGCTTCGCCAGTTGATAGGTAG |
| EgriCXE20 | TTCACCGAATCCACAAGAACAGACC | TGAGGCGACTGGCTGACGATAC |
| EgriCXE21 | GGGACGGATGATGTTCCTGGAAATG | TGCACTACCGCCTTCAATGATAACG |
| EgriCXE22 | TGACATCACAATCATAGGCGGTAGC | AACTTGTAATGCGATGGCTCCTACG |
| EgriCXE24 | CTGTGGACTGACTTTGCTAGGACTG | GTAGTGGTGTACTGACGCCATTCC |
| EgriCXE25 | CAGTGAAACAGGGCAAGTTGAAAGG | CATGGCTTTGGAGGCAGTGGAG |
| EgriCXE26 | CGACCACGCCGACATAACAGATAG | GTCAGGTGGTGCTTCCGTAACG |
| EgriCXE27 | AAGGTGAGCTGCTGAATCTGGTAAC | CCAAGTGGAGGTGCTGCATAAGG |
| EgriCXE29 | CGGAGAAAGTTCTGGAGGTGCTTC | AAACGCTCGCTTCACTGGATGG |
| EgriCXE30 | TTCGTGCGAGTCTGAGCGTAATG | CGACTTCCATCTTCAGCCACTTAGC |
| EgriCXE32 | ACCATTGGACGCAGAAGGCAAG | GCAACGAGACCCACGAAGGAATC |
| EgriCXE33 | CTCGTACTTCACTTACCCAGCGTTG | GGCAAACACCGTAGTCTCCTTCAG |
| EgriCXE34 | TCGTGTTGGCTCTCAAATGGGTTC | TTGCCATCGGAGAAAGCGTCATG |
| EgriCXE36 | CTGTTCCGCAACCAGCAGATCC | TGAGGTAGAGGCAGTCTTCATCTCC |
| EgriCXE37 | CGACTACCGCCTAAGTGCCATTC | CAGGAAGAAGAGTGCCATCGGATTG |
| EgriGSTe1 | CTCAACAGGTCTTGGCTGACATCG | GTGAAGTGGTCCGTCGCTATGAAG |
| EgriGSTe2 | CGTGCCTGTTGACCTAGAAGATGG | GGTACGGCAAGGTTGGAATGTCTC |
| EgriGSTe3 | AGGAGTGGCTGGATCTCGTCTG | CGTCCATCGTGAAGCGGTTGTAG |
| EgriGSTe4 | AAGGAATGTGACCTGTGCCAAGTG | TAGGGCCATGTATCGATGGAAGAGG |
| EgriGSTe5 | GGCCTGTTCCGTTCGGATTATCTC | ATTGAGGGCAGGTTCGAAGTAATGG |
| EgriGSTd1 | GACGACGACGGGTTTCTCTTGAG | AAGCATAGGCGATGATTCACGACAG |
| EgriGSTd2 | TCGGTTCTGGCTCTGAAGATGTTTG | GTTGGAGAAGACACGGCGGAAG |
| EgriGSTd3 | GCCAAGAGTTCGAGGACAAGCG | ATCTCCAGGATCGGCATCTGACC |
| EgriGSTd4 | TTGCTGATGCTACCACCTATGCTTC | CACCGGGTAACTGGGCACTTTC |
| EgriGSTs1 | GTTCGGACGTGATGCTGGACTC | TGCTGTTGATCTCGACTTGCTTCC |
| EgriGSTs2 | TGCCACCTCTATTCACGAATTCACC | GCCACATTGAGAAGCAACATTGACG |
| EgriGSTs3 | GCAGGGCAAGAACCAGGTAATCC | CACAGCGGGCTGGCATTATCTC |
| EgriGSTs4 | GTGACGCTCCCGACTTTGACAG | ATGGCATTGCTCTGGGTGATCTTG |
| EgriGSTt1 | GTGATGCTCTACCCGCCTACAATG | ACGAGGACGCTTCTTTCAGCATAAG |
| EgriGSTo1 | TCAAGGTGCCTTCTACTTTGGTCAG | GATCGGCTATGGTCAATTGGTCTCC |
| EgriGSTu1 | GCTCGTGTCGTCGAACAAGGTG | CTGAGGAGGCAGATATATGGCGAAG |
| GAPDH | CTGCCTCCTACGATGCCATCAAAC | GGACGAGTGCGAGTCACCAATG |

**Table S2. Summary of the sequence assembly of female and male antenna samples** **of the *E. grisescens***

| **Samples** | **Raw**  **Reads** | **Clean**  **Reads** | **Clean Reads rate (%)** | **Clean Bases (Gb)** | **Error rate (%)** | **Q20**  **(%)** | **Q30**  **(%)** | **GC**  **(%)** |
| --- | --- | --- | --- | --- | --- | --- | --- | --- |
| FA_1 | 22343542 | 21733434 | 97.27 | 6.52 | 0.03 | 97.57 | 92.94 | 42.68 |
| FA_2 | 23888193 | 23300358 | 97.54 | 6.99 | 0.03 | 97.66 | 93.28 | 44.79 |
| FA_3 | 22896865 | 22180396 | 96.87 | 6.65 | 0.03 | 97.73 | 93.56 | 44.44 |
| MA_1 | 23436885 | 22713765 | 96.91 | 6.81 | 0.03 | 97.72 | 93.51 | 45.67 |
| MA_2 | 23227820 | 22797995 | 98.15 | 6.84 | 0.03 | 97.65 | 93.29 | 45.79 |
| MA_3 | 23007278 | 22378252 | 97.27 | 6.71 | 0.03 | 97.73 | 93.48 | 45.64 |

Note: FA: female antenna; MA: male antenn

**Table S3. Summary of the *E. grisescens* transcriptomes assembly**

| **Statistics project** | **Transcript** | **Unigene** |
| --- | --- | --- |
| Minimum length (bp) | 301 | 301 |
| Mean length (bp) | 1518 | 1233 |
| Medium length (bp) | 897 | 639 |
| Max length (bp) | 30875 | 30875 |
| N50 (bp) | 2552 | 2170 |
| N90 (bp) | 604 | 462 |
| Total | 124287 | 52856 |

**Table S4. Summary of annotations of the *E. grisescens* transcriptomes**

| **Database** | **Numbers** | **Percentage (%)** |
| --- | --- | --- |
| Annotated in NR | 19118 | 36.16 |
| Annotated in NT | 15500 | 29.32 |
| Annotated in KO | 7321 | 13.85 |
| Annotated in SwissProt | 11512 | 21.77 |
| Annotated in PFAM | 14325 | 27.10 |
| Annotated in GO | 14323 | 27.09 |
| Annotated in KOG | 6119 | 11.57 |
| Annotated in all Databases | 3097 | 5.85 |
| Annotated in at least one Database | 27638 | 52.28 |
| Total unigenes | 52856 | 100 |

**Table S6. Catalytic motifs of the *E. grisescens* CXEs**

| **Gene name** | **Catalytic motifs (amino acids)** | | | |
| --- | --- | --- | --- | --- |
|  | **GxSxG version** | **E** | **H** | **Oxyanion hole version** |
| EgriCXE1 | GESAG | + | + | GGC |
| EgriCXE2 | GESWG | + | + | GGG |
| EgriCXE3 | GESAG | + | + | GGA |
| EgriCXE4 | GESWG | + | + | GGG |
| EgriCXE5 | GCSAG | + | + | AGG |
| EgriCXE6 | GHSSA | + | + | AEE |
| EgriCXE8 | GHSAG | + | + | GGG |
| EgriCXE9 | GSSSG | + | + | - |
| EgriCXE11 | GVSAG | + | + | GGG |
| EgriCXE13 | GCSAG | + | + | GGA |
| EgriCXE15 | GQSAG | + | + | - |
| EgriCXE17 | GESAG | + | + | GGG |
| EgriCXE18 | GQSAG | + | + | GGA |
| EgriCXE19 | GHDAG | + | + | GGG |
| EgriCXE20 | GESAG | + | + | GGG |
| EgriCXE21 | GGSAG | + | + | GGA |
| EgriCXE22 | GGSAG | + | + | GGA |
| EgriCXE24 | GESAG | + | + | GGA |
| EgriCXE25 | GESAG | + | + | GGA |
| EgriCXE26 | GCSAG | + | + | GGG |
| EgriCXE27 | GESAG | + | + | GGG |
| EgriCXE29 | GESSG | + | + | GGG |
| EgriCXE30 | GESAG | + | + | GGG |
| EgriCXE32 | GQGSG | + | + | GGN |
| EgriCXE33 | GESAG | + | + | GGG |
| EgriCXE34 | GSSSG | + | + | - |
| EgriCXE36 | GISAG | + | + | GGG |
| EgriCXE37 | GHSAG | + | + | - |

**Table S8. Accession numbers of the insects CXEs used in the phylogenetic analysis**

| **Species** | **CXE name** | **Accession number** |
| --- | --- | --- |
| *Ectropis obliqua* | EoblCXE1 | KX015843 |
|  | EoblCXE2 | KX015844 |
|  | EoblCXE3 | KX015845 |
|  | EoblCXE4 | KX015846 |
|  | EoblCXE5 | KX015847 |
|  | EoblCXE6 | KX015848 |
|  | EoblCXE7 | KX015849 |
|  | EoblCXE8 | KX015850 |
|  | EoblCXE9 | KX015851 |
|  | EoblCXE10 | KX015852 |
|  | EoblCXE11 | KX015853 |
|  | EoblCXE12 | KX015854 |
|  | EoblCXE13 | KX015855 |
|  | EoblCXE14 | KX015856 |
|  | EoblCXE15 | KX015857 |
|  | EoblCXE16 | KX015858 |
|  | EoblCXE17 | KX015859 |
|  | EoblCXE18 | KX015860 |
|  | EoblCXE19 | KX015861 |
|  | EoblCXE20 | KX015862 |
|  | EoblCXE21 | KX015863 |
|  | EoblCXE22 | KX015864 |
|  | EoblCXE23 | KX015865 |
|  | EoblCXE24 | KX015866 |
|  | EoblCXE25 | KX015867 |
|  | EoblCXE26 | KX015868 |
|  | EoblCXE27 | KX015869 |
|  | EoblCXE28 | KX015870 |
|  | EoblCXE29 | KX015871 |
|  | EoblCXE30 | KX015872 |
|  | EoblCXE31 | KX015873 |
|  | EoblCXE32 | KX015874 |
|  | EoblCXE33 | KX015875 |
|  | EoblCXE34 | KX015876 |
|  | EoblCXE35 | KX015877 |
| *Spodoptera littoralis* | SlittCXE2 | FJ652445 |
|  | SlittCXE3 | FJ652446 |
|  | SlittCXE4 | FJ652447 |
|  | SlittCXE5 | FJ652448 |
|  | SlittCXE6 | FJ652449 |
|  | SlittCXE7 | FJ652450 |
|  | SlittCXE8 | FJ652451 |
|  | SlittCXE9 | FJ652452 |
|  | SlittCXE10 | FJ652453 |
|  | SlittCXE11 | FJ652454 |
|  | SlittCXE12 | FJ652455 |
|  | SlittCXE13 | FJ652456 |
|  | SlittCXE14 | FJ652457 |
|  | SlittCXE15 | FJ652458 |
|  | SlittCXE16 | FJ652459 |
|  | SlittCXE17 | FJ652460 |
|  | SlittCXE18 | FJ652461 |
|  | SlittCXE19 | FJ652462 |
|  | SlittCXE20 | FJ652463 |
| *Plodia interpunctella* | PintCXE1 | MK864061 |
|  | PintCXE2 | MK864062 |
|  | PintCXE3 | MK864063 |
|  | PintCXE4 | MK864064 |
|  | PintCXE8 | MK864068 |
|  | PintCXE9 | MK864069 |
|  | PintCXE11 | MK864071 |
|  | PintCXE12 | MK864072 |
|  | PintCXE13 | MK864073 |
|  | PintCXE14 | MK864074 |
|  | PintCXE15 | MK864075 |
|  | PintCXE16 | MK864076 |
|  | PintCXE17 | MK864077 |
|  | PintCXE18 | MK864078 |
|  | PintCXE19 | MK864079 |
|  | PintCXE20 | MK864080 |
|  | PintCXE21 | MK864081 |
|  | PintCXE23 | MK864083 |
|  | PintCXE25 | MK864085 |
| *Spodoptera exigua* | SexiCXE5 | HQ116561 |
|  | SexiCXE10 | JF728805 |
|  | SexiCXE11 | JF728804 |
|  | SexiCXE13 | HQ116560 |
|  | SexiCXE14 | JF728803 |
|  | SexiCXE17 | HQ116559 |
|  | SexiCXE18 | JF728802 |
|  | SexiCXE20 | HQ116562 |
| *Spodoptera litura* | SlituCXE13 | HQ116556 |
|  | SlituCXE17 | HQ116558 |
|  | SlituCXE18 | HQ116557 |
| *Sesamia inferens* | SinfCXE1 | KF960776 |
|  | SinfCXE3 | KF960778 |
|  | SinfCXE5 | KF960779 |
|  | SinfCXE6 | KF960780 |
|  | SinfCXE9 | KF960781 |
|  | SinfCXE10 | KF960782 |
|  | SinfCXE11 | KF960783 |
|  | SinfCXE12 | KF960784 |
|  | SinfCXE13 | KF960785 |
|  | SinfCXE14 | KF960786 |
|  | SinfCXE16 | KF960787 |
|  | SinfCXE18 | KF960788 |
|  | SinfCXE19 | KF960789 |
|  | SinfCXE20 | KF960790 |
|  | SinfCXE26 | KF960791 |
|  | SinfCXE28 | KF960792 |

**Table S9. Accession numbers of the insects GSTs used in the phylogenetic analysis**

| **Species** | **CXE name** | **Accession number** |
| --- | --- | --- |
| *Plodia interpunctella* | PintGSTd1 | MZ410553 |
|  | PintGSTd2 | MZ410560 |
|  | PintGSTd3 | MZ410545 |
|  | PintGSTe1 | MZ410556 |
|  | PintGSTe2 | MZ410551 |
|  | PintGSTo1 | MZ410557 |
|  | PintGSTo2 | MZ410559 |
|  | PintGSTo3 | MZ410550 |
|  | PintGSTs1 | MZ410548 |
|  | PintGSTs2 | MZ410552 |
|  | PintGSTt1 | MZ410547 |
|  | PintGSTu1 | MZ410554 |
| *Plutella xylostella* | PxylGSTd1 | You et al., 2015 |
|  | PxylGSTd2 |  |
|  | PxylGSTd3 |  |
|  | PxylGSTd4 |  |
|  | PxylGSTd5 |  |
|  | PxylGSTe1 |  |
|  | PxylGSTe2 |  |
|  | PxylGSTe3 |  |
|  | PxylGSTe4 |  |
|  | PxylGSTe5 |  |
|  | PxylGSTo1 |  |
|  | PxylGSTo2 |  |
|  | PxylGSTo3 |  |
|  | PxylGSTo4 |  |
|  | PxylGSTo5 |  |
|  | PxylGSTs1 |  |
|  | PxylGSTs2 |  |
|  | PxylGSTt1 |  |
|  | PxylGSTu1 |  |
| *Cydia pomonella* | CpomGSTd2 | KX500029 |
|  | CpomGSTd3 | KX500030 |
|  | CpomGSTd4 | KX500031 |
|  | CpomGSTe1 | KX500032 |
|  | CpomGSTe2 | KX500033 |
|  | CpomGSTe3 | KX500034 |
|  | CpomGSTo1 | KX500038 |
|  | CpomGSTo2 | KX500039 |
|  | CpomGSTu1 | KX500040 |
| *Bombyx mori* | BmorGSTd1 | NP_001037183.1 |
|  | BmorGSTd2 | NP_001036974.1 |
|  | BmorGSTd3 | NP_001037546.1 |
|  | BmorGSTe1 | NP_001037197.1 |
|  | BmorGSTe2 | NP_001345908.1 |
|  | BmorGSTe3 | NP_001108466.1 |
|  | BmorGSTe4 | NP_001108460.1 |
|  | BmorGSTe5 | NP_001108464.1 |
|  | BmorGSTe6 | NP_001108465.1 |
|  | BmorGSTo1 | NP_001040131.1 |
|  | BmorGSTo2 | NP_001037406.1 |
|  | BmorGSTo3 | NP_001040435.1 |
|  | BmorGSTo4 | NP_001108461.1 |
|  | BmorGSTs1 | NP_001037077.1 |
|  | BmorGSTs2 | NP_001036994.1 |
|  | BmorGSTt1 | NP_001108463.1 |
|  | BmorGSTu1 | NP_001108462.1 |
| *Chilo suppressalis* | CsupGSTd1 | KP938857 |
|  | CsupGSTd2 | KP938858 |
|  | CsupGSTd3 | KP938859 |
|  | CsupGSTd4 | KP938860 |
|  | CsupGSTe1 | KP938861 |
|  | CsupGSTe2 | KP938862 |
|  | CsupGSTe3 | KP938863 |
|  | CsupGSTo1 | KP938864 |
|  | CsupGSTo2 | KP938865 |
|  | CsupGSTo3 | KP938866 |
|  | CsupGSTo4 | KP938867 |
|  | CsupGSTs1 | GU453917 |
|  | CsupGSTs2 | KP938868 |
|  | CsupGSTt1 | KP938869 |
|  | CsupGSTu1 | KP938871 |
| *Acyrthosiphon pisum* | ApisGSTd1 | [NP_001280269](https://www.ncbi.nlm.nih.gov/protein/NP_001280269.1?report=genbank&log$=protalign&blast_rank=1&RID=Y52JA6KK013).1 |
|  | ApisGSTd3 | [XP_008186556.2](https://www.ncbi.nlm.nih.gov/protein/XP_008186556.2?report=genbank&log$=protalign&blast_rank=1&RID=Y52T11PJ016) |
|  | ApisGSTd7 | NP_001156274.1 |
|  | ApisGSTd8 | [XP_029345031.1](https://www.ncbi.nlm.nih.gov/protein/XP_029345031.1?report=genbank&log$=protalign&blast_rank=1&RID=Y531Z0RZ013) |
|  | ApisGSTd11 | [XP_029345032.1](https://www.ncbi.nlm.nih.gov/protein/XP_029345032.1?report=genbank&log$=protalign&blast_rank=1&RID=Y534K2DY013) |
|  | ApisGSTd15 | [AFM57708.1](https://www.ncbi.nlm.nih.gov/protein/AFM57708.1?report=genbank&log$=protalign&blast_rank=1&RID=Y537WNYZ016) |
|  | ApisGSTo1 | [NP_001155757.1](https://www.ncbi.nlm.nih.gov/protein/NP_001155757.1?report=genbank&log$=protalign&blast_rank=1&RID=Y53AGF62013) |
|  | ApisGSTs1 | [NP_001156063.1](https://www.ncbi.nlm.nih.gov/protein/NP_001156063.1?report=genbank&log$=protalign&blast_rank=1&RID=Y53D0FTD013) |
|  | ApisGSTs2 | [NP_001313601.1](https://www.ncbi.nlm.nih.gov/protein/NP_001313601.1?report=genbank&log$=protalign&blast_rank=1&RID=Y53GKHDC016) |
|  | ApisGSTs3 | [NP_001156113.1](https://www.ncbi.nlm.nih.gov/protein/NP_001156113.1?report=genbank&log$=protalign&blast_rank=1&RID=Y53JT7J2016) |
|  | ApisGSTs4 | [NP_001280393.1](https://www.ncbi.nlm.nih.gov/protein/NP_001280393.1?report=genbank&log$=protalign&blast_rank=1&RID=Y53P6WH7016) |
|  | ApisGSTs6 | [XP_001952040.2](https://www.ncbi.nlm.nih.gov/protein/XP_001952040.2?report=genbank&log$=protalign&blast_rank=1&RID=Y53SNEGW016) |
|  | ApisGSTt1 | [NP_001156289.1](https://www.ncbi.nlm.nih.gov/protein/NP_001156289.1?report=genbank&log$=protalign&blast_rank=1&RID=Y53XG4NZ016) |
|  | ApisGSTt2 | [BAH70628.1](https://www.ncbi.nlm.nih.gov/protein/BAH70628.1?report=genbank&log$=protalign&blast_rank=1&RID=Y540HWEX016) |
| *Drosophila melanogaster* | DmelGSTd1 | NP_001034042.1 |
|  | DmelGSTd2 | NP_524912.1 |
|  | DmelGSTd3 | NP_788656.1 |
|  | DmelGSTd4 | NP_524913.1 |
|  | DmelGSTd5 | NP_524914.1 |
|  | DmelGSTd6 | NP_524915.1 |
|  | DmelGSTd7 | NP_525114.1 |
|  | DmelGSTd8 | NP_524916.1 |
|  | DmelGSTd9 | NP_650181.1 |
|  | DmelGSTd10 | NP_652713.1 |
|  | DmelGSTe1 | NP_611323.1 |
|  | DmelGSTe2 | NP_611324.1 |
|  | DmelGSTe3 | NP_611325.1 |
|  | DmelGSTe4 | NP_611326.1 |
|  | DmelGSTe5 | NP_611327.1 |
|  | DmelGSTe6 | NP_611328.1 |
|  | DmelGSTe7 | NP_611329.1 |
|  | DmelGSTe8 | NP_611330.1 |
|  | DmelGSTe10 | NP_611322.1 |
|  | DmelGSTs1 | NP_001261040.1 |
| *Anopheles gambiae* | AgamGSTd3 | AF513638 |
|  | AgamGSTd4 | AF513635 |
|  | AgamGSTd5 | AF513634 |
|  | AgamGSTd6 | AF513636 |
|  | AgamGSTd7 | AF071161 |
|  | AgamGSTd10 | AF515527 |
|  | AgamGSTd12 | AF316638 |
|  | AgamGSTd15 | [XP_307500.3](https://www.ncbi.nlm.nih.gov/protein/XP_307500.3?report=genbank&log$=prottop&blast_rank=1&RID=Y50AH3PD016) |
|  | AgamGSTd16 | [XP_306603](https://www.ncbi.nlm.nih.gov/protein/XP_306603.3?report=genbank&log$=prottop&blast_rank=1&RID=Y50E8CD5016).3 |
|  | AgamGSTe1 | AF316635 |
|  | AgamGSTe2 | AF316636 |
|  | AgamGSTe4 | AY070254 |
|  | AgamGSTe5 | AY070255 |
|  | AgamGSTe6 | AY070256 |
|  | AgamGSTe7 | AF491816 |
|  | AgamGSTo1 | AY255856 |
|  | AgamGSTs1 | AF513639 |
|  | AgamGSTt1 | AF515526 |
|  | AgamGSTt2 | AF515525 |
|  | AgamGSTu1 | AF515521 |
| *Tribolium astaneum* | TcasGSTd1 | XP_974273.1 |
|  | TcasGSTo1 | XP_971118.1 |
|  | TcasGSTo2 | XP_971184.1 |
|  | TcasGSTt1 | XP_968617.1 |
|  | TcasGSTo3 | Shi et al., 2012 |
|  | TcasGSTs1 |  |
|  | TcasGSTs2 |  |
|  | TcasGSTs3 |  |
|  | TcasGSTs4 |  |
|  | TcasGSTs5 |  |
|  | TcasGSTs6 |  |
|  | TcasGSTs7 |  |
| *Grapholita molesta* | GmolGSTD1 | MG696891 |
| *Sitophilus zeamais* | SzeaGSTd1 | MW390709 |

# Supplementary Figures

**Figure S1 Amino acid sequence structure analysis of EgriGSTs**
